# Antigenic Characterization of H1N1 Influenza Viruses That Circulated During the 2019–2020 Season in Philadelphia, Pennsylvania

**DOI:** 10.1111/irv.70104

**Published:** 2025-06-01

**Authors:** Lydia M. Mendoza, Elizabeth M. Anderson, Ashley Sobel Leonard, Alexander G. McFarland, Jordan T. Ort, Nicole Tanenbaum, Afeesat Durosinmi, Frederic D. Bushman, Laurel J. Glaser, Irving Nachamkin, Scott E. Hensley

**Affiliations:** ^1^ Department of Microbiology, Perelman School of Medicine University of Pennsylvania Philadelphia Pennsylvania USA; ^2^ Division of Infectious Diseases Children's Hospital of Philadelphia Philadelphia Pennsylvania USA; ^3^ Department of Pathology and Laboratory Medicine, Perelman School of Medicine University of Pennsylvania Philadelphia Pennsylvania USA

**Keywords:** antibody, antigenic drift, influenza

## Abstract

**Background:**

Multiple clades of H1N1 influenza A viruses (IAVs) circulated during the 2019–2020 season. Here, we completed serological assays to determine the specificities of serum antibodies from humans infected with viruses from different H1N1 clades during the 2019–2020 season.

**Methods:**

We collected nasopharyngeal (NP) swabs and serum from influenza‐infected individuals who received care within the University of Pennsylvania Health System (UPHS). We sequenced H1N1 viruses from NP swabs and completed hemagglutination inhibition assays using serum and viruses from different H1N1 clades that we identified from NP swabs. We also collected serum samples from influenza B virus (IBV)–infected patients at UPHS, allowing us to examine antibody titers associated with H1N1 versus IBV infection.

**Results:**

Sequence analyses revealed that most IAV‐infected individuals were infected with clade 6B.1A.5a.1 and 6B.1A.5a.2 H1N1 viruses that possessed substitutions at major antigenic sites of hemagglutinin. We found that antibodies from both H1N1‐ and IBV‐infected individuals recognized the 6B.1A.1 H1N1 vaccine component of the 2019–2020 vaccine more efficiently compared to the circulating 6B.1A.5a.1 and 6B.1A.5a.2 H1N1 viruses. Patients infected with 6B.1A.5a.2 clade H1N1 viruses had significantly higher titers against the vaccine strain virus, suggesting that the 6B.1A.5a.2 virus evaded antibodies elicited from previous vaccinations or infections.

**Conclusions:**

These studies suggest that most individuals, irrespective of whether they were infected with H1N1 virus or IBV during the 2019–2020 season, possessed antibodies that poorly reacted to circulating H1N1 strains.

## 1 Background

The burden of seasonal influenza virus varies year to year but is consistently a threat to human health with on average between 9.3 and 41 million illnesses, 100,000 to 710,000 hospitalizations, and between 4900 and 51,000 deaths in the United States each year [1]. Influenza vaccines are updated annually because both influenza A viruses (IAVs) and influenza B viruses (IBVs) continuously acquire substitutions within the hemagglutinin (HA) glycoprotein that affect antigenicity. Current influenza vaccines include immunogens from one H1N1 virus, one H3N2 virus, and one IBV [2]. Surveillance activities around the globe are important for identifying antigenically distinct viruses as they emerge.

The 2019–2020 Northern Hemisphere influenza season was characterized by an initial wave of IBVs followed by the predominant spread of multiple clades of H1N1 IAVs [3]. The H1N1 vaccine component for this season was A/Brisbane/2/2018 of clade 6B.1A.1 [4, 5]. Multiple clades of H1N1 viruses circulated during the 2019–2020 season that possessed substitutions within previously defined antigenic regions of the HA protein [6]. The HA genes of viruses circulating worldwide during this season fell within the 6B.1A.5 and 6B.1A.7 phylogenetic clades [7]. The 6B.1A.5 clade is split into 6B.1A.5a and 6B.1A.5b clades. Viruses within the 6B.1A.5b clade possessed a K160M substitution within antigenic site Sa and a E235D substitution within antigenic site Ca1 [8]. Most viruses circulating during the 2019–2020 season fell within the 6B.1A.5a subclade, which featured HA amino acid substitutions N129D, T185I and N260D. The 6B.1A.5a subclade is further split into 6B.1A.5a.1, with HA1 substitutions at D187A and Q189E within antigenic site Sb, and 6B.1A.5a.2, which possessed an HA1 substitution within antigenic site Sa at N156K [5, 7]. Some antigenic substitutions present in H1N1 clades in the 2019–2020 season, including substitutions in the 130 loop (K130N in 6B.1A.5b and 6B.1A.5a.2) and the 190 helix (D187A and Q189E in 6B.1A.5a.1), have also been reported to affect HA receptor binding as they are located near the HA receptor binding site [9, 10]. Key substitutions in the H1 protein are summarized in Table 1.

**TABLE 1:** Summary of substitutions in the H1 protein of H1N1 virus clades relative to the 2019–2020 vaccine strain virus.

H1N1 clade	Amino acid substitutions	Antigenic sites
6B.1A.1	—	—
6B.1A.5b	K160M, E235D, K130N	K160M in site Sa, E235D in site Ca1
6B.1A.5a	N129D, T185I, N260D	T185I in site Sb
6B.1A.5a.1	N129D, T185I, N260D, D187A, Q189E	T185I in site Sb, D187A and Q189E in site Sb
6B.1A.5a.2	N129D, T185I, N260D, N156K, K130N	T185I in site Sb, N156K in site Sa

*Note:* This table summarizes the clade designations, key amino acid changes, and antigenic sites impacted by these amino acid changes. The vaccine strain clade 6B.1A.1 is included as a reference.

The overall vaccine effectiveness (VE) for the H1N1 component of the 2019–2020 vaccine was 30% [11], which is lower than most years since the emergence of the pandemic H1N1 virus in 2009 [12]. The relatively low VE during the 2019–2020 season has been attributed to the presence of HA antigenic mutations in clade 6B.1A.5a.1 and 6B.1A.5a.2 H1N1 viruses [13]. Post‐infection ferret antisera raised against an A/Brisbane/02/2018‐like virus do not efficiently recognize viruses with amino acid substitutions at HA1 residues 155 or 156 [14, 15, 16]. Ferret antisera against an A/Brisbane/02/2018‐like virus reacted poorly to a 6B.1A.5a.2 clade virus, with a more subtle reduction to a 6B.1A.5a.1 clade virus [16]. Human post‐vaccination serum from the 2017 to 2018 influenza season recognized an A/Brisbane/02/2018‐like reference virus more efficiently than 6B.1A.5a.1 and 6B.1A.5a.2 clade viruses possessing the 187A + 189E and 156K HA1 substitutions [15]. Studies that examined infections at the clade level during the 2019–2020 season determined that VE was 41% against 6B.1A.5a.1‐like viruses and only 7% against 6B.1A.5a.2‐like viruses [11]. Studies evaluating protection against hospitalization found similar results where VE was 59% for 6B.1A.5a.1‐like viruses and not effective at all for 6B.1A.5a.2 viruses [17]. Taken together, these studies suggest that antigenic substitutions, especially those in the 6B.1A.5a.2 viruses, contributed to low VE during the 2019–2020 influenza season.

In this study, we analyzed paired nasopharyngeal (NP) swabs and serum samples from H1N1‐ and IBV‐infected patients collected at the Hospital of the University of Pennsylvania (HUP) and affiliated outpatient facilities within the University of Pennsylvania Health System (UPHS) during the 2019–2020 season. We sequenced viruses and identified individuals infected with different H1N1 clades. We tested whether individuals infected with different H1N1 viruses possessed serum antibodies with unique specificities and if these antibody levels were lower compared to patients infected with IBV. We found that most individuals in our study, irrespective of whether they were infected with H1N1 or IBV, possessed antibodies that reacted weakly to antigenically drifted H1N1 viruses that circulated during the 2019–2020 season.

## 2 Methods

### 2.1 Human Samples

All experiments involving residual samples from human subjects were reviewed and approved by the University of Pennsylvania Institutional Review Board (Protocol No. 834238). Residual serum samples were collected from the core laboratory and residual NP swabs from the clinical microbiology laboratory at HUP from 35 IAV‐infected individuals and 33 IBV‐infected individuals from December 2019 through February 2020. The Cepheid Xpert Xpress Flu/RSV system was used to test NP swabs and identify IAV and IBV infections. Through next‐generation sequencing, we found that 6 of the IAV cases were H3N2 virus infections, and therefore, their sera were not included in our analyses. In total, we included samples from 29 H1N1‐infected patients (age range 21–70 years, mean 44.1 years) and 33 IBV‐infected patients (age range 21–92, mean 35.6 years) in our analyses. Serum samples were treated with receptor destroying enzyme (RDE, Accurate Chemical No. YCC340‐122) followed by heat inactivation at 55°C for 30 min. The serum samples were pre‐incubated with red blood cells (RBCs) by combining RDE‐treated serum volume with 1.1× the volume of 10% RBCs in PBS. This mixture was incubated at 4°C for 1 h before centrifuging the solution and removing the serum supernatant. Serum was stored at 4°C until use in the HAI assay.

### 2.2 Sequencing Viruses From NP Swabs

Influenza viral RNA was extracted from NP swab samples using the QIAGEN QIAamp Viral RNA Mini Kit (QIAGEN 52904). Extracted RNA was reverse‐transcribed and amplified with the Superscript III One‐Step RT‐PCR Kit with Platinum Taq High Fidelity DNA Polymerase (Fisher No. 12574‐035) using universal influenza primers Uni12/Inf1 (GGGGGGAGCAAAAGCAGG), Uni12/Inf3 (GGGGGGAGCGAAAGCAGG), and Uni13/Inf1 (CGGGTTATTAGTAGAAACAAGG) to amplify all eight segments of the influenza genome. Reactions consisted of 12.5 μL 2× buffer, 0.2 μL Uni12/Inf1 primer, 0.3 μL Uni12/Inf3 primer, 0.5 μL Uni13/Inf1 primer, 0.5 μL Taq HiFi DNA polymerase, 6 μL UltraPure DEPC‐Treated Water (Thermo Fisher #750023), and 5 μL of viral RNA extracted from NP swabs. The thermocycler protocol was as follows: 42°C for 60 min, 94°C for 2 min, followed by 5 cycles of 30 s at 94°C, 30 s at 44°C, and 3 min at 68°C, followed by 28 cycles of 30 s at 94°C, 30 s at 57°C, and 3 min at 68°C. The resulting cDNA was purified with AMPure XP magnetic beads (Beckman Coulter No. A63881) and prepared for sequencing with the Illumina DNA Prep (M) Tagmentation (Illumina No. 20060059 and 20018705) kits and IDT for Illumina DNA/RNA UD indexes (No. 20027213). The DNA concentration of each sample was normalized to 2 ng using Quant‐IT PicoGreen (Thermo No. PLL496), and all samples were combined. The pooled cDNA library was purified with AMPure XP magnetic beads. The concentration of the pooled library was determined using the Qubit 1× dsDNA high‐sensitivity assay kit (Thermo No. Q33230) and diluted to 2 nM. The library was sequenced using the NextSeq 500/550 High Output Kit (300 cycles) (Illumina No. 20024908) and NextSeq 500 machine (Illumina). Each sequencing run included positive control viruses created by reverse genetics. Our H3N2 positive control possessed HA and neuraminidase (NA) segments from A/Cambodia/e0826360/2020 and internal gene segments from A/Puerto Rico/8/1934. We also included an H6N1 positive control with HA from A/Turkey/Massachusetts/3740/1965, NA from A/California/07/2009 (H1N1), and internal gene segments from A/Puerto Rico/8/1934. A water negative control was included for each sequencing run.

### 2.3 Sequence Processing

Samples were demultiplexed using custom bash scripts and processed using FluPipeline. FluPipeline is a command line program that uses short read data to generate consensus sequences through alignment to a reference genome. Our sequence preprocessing steps and processing with FluPipeline were performed as previously described [18]. FluPipeline is available on GitHub (https://github.com/agmcfarland/FluPipeline).

Sequence post‐processing was completed with custom R scripts available on GitHub (https://github.com/HensleyLab‐UPENN/HUP_Influenza). Portions of the code for the R scripts for data processing and statistical analysis were optimized with the assistance of the OpenAI GPT‐4 language model (OpenAI). Packages used in post‐processing analysis are listed in the Readme file. Packages used for statistical analysis and manipulation of genomic information used for the above analyses include tidyverse v2.0.0 [19], Rsamtools v2.14.0 [20], Biostrings v2.66.0 [21], GenomicRanges v1.50.2 [22], dplyr v1.1.2 [23], seqinr v4.2.30 [24], stringr v1.5.0 [25], phylotools v0.2.2 [26], stringi v1.7.12 [27], ggplot2 v3.4.2 [28], tidysq v1.2.0 [29], patchwork v1.1.2 [30], purrr v1.0.1 [31], gridExtra v2.3 [32], ShortRead v1.56.1 [33], and data.table v1.14.8 [34]. The FASTQ files for the influenza isolates analyzed in this study are available from the NCBI SRA database in BioProject PRJNA1256457.

During post‐processing, we applied additional quality control criteria to ensure adequate coverage and sequence reproducibility. For coverage, we required that samples have > 40 reads for ≥ 95% of the genome. Individual sites with coverage < 40 reads were masked. To ensure reproducibility, we required that all samples have technical replicates, that is, replicates from separate reverse transcription and amplification reactions, and that the consensus sequences for these replicates were identical. We allowed exceptions for disagreements between the replicate consensus sequences if the mismatch occurred within either the first or last 20 bp of the gene segment. The post‐processing script produced FASTA files containing the sequences of all samples that passed QC for all eight influenza segments. The FASTA file of the HA sequences was used for the construction of a phylogenetic tree to show the HA genetic variation present in the sample population.

### 2.4 Cell Culture

Human embryonic kidney (HEK) 293T cells and Madin–Darby canine kidney (MDCK) cells were purchased from American type cell culture (ATCC) and were grown in DMEM (Corning MT10‐013‐CM) and MEM (Corning MT10‐010‐CM), respectively, with 10% fetal bovine serum (Sigma F2442‐500ML). Cells were cultured at 37°C with 5% carbon dioxide.

### 2.5 Viruses

We used reverse genetics to generate influenza viruses for this study. The HA and NA genes from each virus strain were cloned into the pHW2000 vector. These plasmids were transfected into a co‐culture of 293T and MDCK cells with A/Puerto Rico/8/1934 internal genes using Lipofectamine 2000 (Thermo Fisher 11668019) and Opti‐MEM (Thermo Fisher 31985070). Transfection supernatants were collected 3 days later and used to infect 10‐day‐old, fertilized laboratory‐grade chicken eggs (Charles River) for 48 h at 37°C. Allantoic fluid from the infected eggs was collected 48 h later. The allantoic fluid was centrifuged to remove cells and frozen at −80°C. The HA and NA genes of egg expanded viruses were confirmed by Sanger sequencing.

### 2.6 Hemagglutination Inhibition Assay

Two milliliters of Turkey RBCs (Lampire No. 7209401) were combined with 12 mL of phosphate‐buffered saline (PBS, Corning No. 7209401) and centrifuged at 2000 rpm for 5 min. Supernatant was aspirated, and the RBC pellet was resuspended in 12 mL of PBS and centrifuged. This process was repeated for a total of two washes. 200 μL of the RBC pellet was combined with 9.8 mL of PBS with 20 nM oseltamivir to create a 2% RBC solution. Oseltamivir was included in the hemagglutination inhibition (HAI) assays to prevent NA cleavage of sialic acid on RBCs. Serum samples were serially diluted 1:2 in PBS with oseltamivir using a 96‐well U‐bottom plate. 12.5 μL of 2% RBCs were added to each well and incubated for 1 h. The 96‐well plates were suspended vertically for 1 min to allow for teardrop shapes of RBCs to form prior to imaging and scoring. Replicate experiments were conducted on separate days, and the geometric mean of the replicates was calculated to determine the HAI titer.

### 2.7 Phylogenetics

To contextualize the HA sequences of HUP viral isolates with other viruses that circulated in the United States during the 2019–2020 season, we constructed a phylogenetic tree using the Nextstrain pipeline [5]. All available full‐length HA sequences from human H1N1 isolates collected in the United States between October 1, 2019, and April 30, 2020, were downloaded from GISAID (gisaid.org), along with HA sequences of viral strains used for reverse genetics. An acknowledgment list including accession numbers, originating labs, and submitting labs for all GISAID sequences is provided in Table S1. To build the tree, the US sequences were randomly subsampled to 40 isolates per month, and reverse genetics strains and consensus sequences from HUP isolates were included. Sequences were aligned with MAFFT [35] and divergence phylogenies were created using IQ‐TREE 2 [36] using the general time reversible (GTR) model. Tree visualization was performed using Baltic (github.com/evogytis/baltic).

## 3 Results

### 3.1 Clinical Samples Analyzed in This Study

**FIGURE 1:**
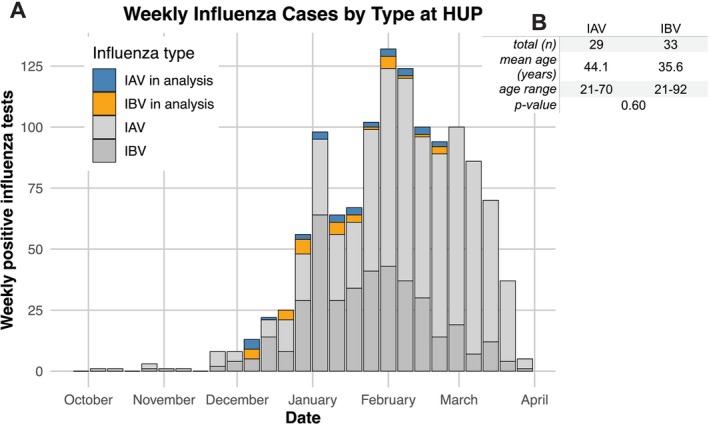
IAV and IBV circulation in Philadelphia during the 2019–2020 season. (A) Shown are the total number of influenza positive tests per week at the Hospital of the University of Pennsylvania (HUP) from October 2019 to April 2020. Blue and orange indicate samples analyzed in this study. (B) A summary of the number and age of IAV and IBV‐infected individuals analyzed in this study.

IAVs and IBVs circulated widely in the Philadelphia region during the 2019–2020 season (Figure 1A). Influenza virus cases abruptly decreased as mitigation measures were put into place in response to the COVID‐19 pandemic [37]. This pattern of viral circulation was observed throughout the United States during the 2019–2020 season. Most IAVs that circulated nationally were of the H1N1 subtype [3]. We carried out experiments to determine whether individuals infected with different clades of H1N1 possess unique antibody repertoires compared to individuals infected with IBVs. We collected NP swab and serum samples from 29 H1N1‐infected individuals as well as serum from 33 IBV‐infected individuals that sought care within the UPHS whose clinical samples were sent to HUP for testing. H1N1‐infected individuals were slightly older (mean age 44.1 years old) compared to IBV‐infected individuals (mean age 35.6 years old), but this age difference was not statistically significant (*p* = 0.60, unpaired *t*‐test) (Figure 1B).

**FIGURE 2:**
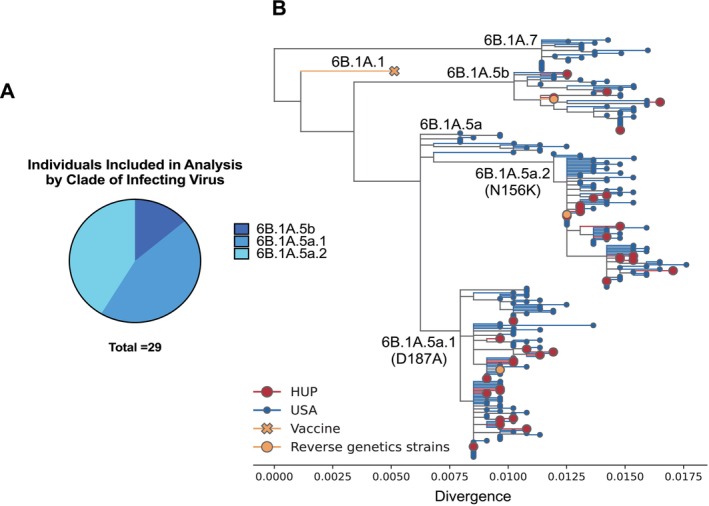
Diverse H1N1 clades circulated in Philadelphia during the 2019–2020 season. (A) Three different clades of H1N1 influenza virus were detected in the population of individuals that were included in this study. (B) Phylogenetic reconstruction of HA sequences from HUP NP swab isolates (red circles), with contextual H1N1 sequences from the USA during the 2019–2020 influenza season (blue circles). The clade 6B.1A.1 vaccine strain (A/Brisbane/02/2018) is depicted as a yellow “×” and representative strains for clades 6B.1A.5b (A/Delaware/37/2019), 6B.1A.5a.2 (A/Victoria/2570/2019), and 6B.1A.5a.1 (A/Hawaii/70/2019) used for reverse genetics viruses are depicted as yellow circles. Clades are annotated, and notable antigenic HA substitutions present in 6B.5a.2 and 6B.5a.1 are displayed in parentheses. Divergence between the HA sequences is displayed on the x‐axis as nucleotide substitutions per site. Viruses from clades 6B.1A.7 and 6B.1A.5a were present at the national level within the United States, but not in our HUP isolates.

We completed next‐generation sequencing of the entire influenza genome and used the HA sequence to determine the viral clade for H1N1 infections identified in this study. Most individuals were infected with either a 6B.1A.5a.1 or 6B.1A.5a.2 clade of H1N1 virus, and a smaller group of individuals were infected with a virus of the 6B.1A.5b clade (Figure 2A). We completed a phylogenetic reconstruction using HA sequences from the HUP isolates in our study, along with 200 HA sequences from H1N1 viruses collected in the United States during the 2019–2020 influenza season (Figure 2B). The tree tips of the HUP samples we sequenced fell within the same major clades as contemporaneous sequences from the United States. The 6B.1A.5a.1 and 6B.1A.5a.2 clades of virus were identified most often in both our samples and in the representative viruses that we analyzed. Therefore, the virus clades observed in our study are mostly representative of the viruses that were circulating in the United States. However, we did not detect the 6B.1A.5a and 6B.1A.7 clades of virus, which circulated at low levels.

### 3.2 Comparison of Sera From H1N1‐Infected and IBV‐Infected Individuals

**FIGURE 3:**
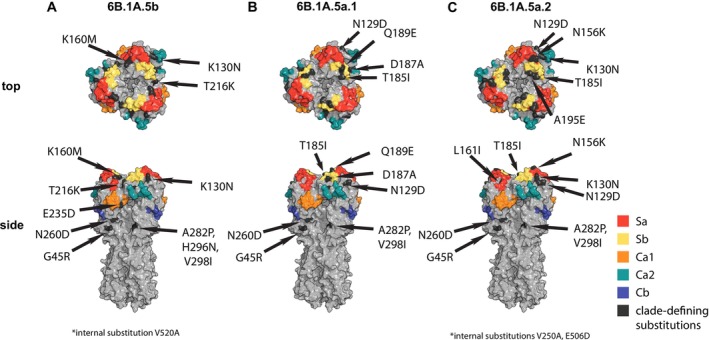
H1N1 viruses in NP swab isolates possessed HA substitutions relative to the 2019–2020 H1N1 vaccine strain. Top and side views of the HA protein (PDB 4LXV) [38] highlighting clade‐defining substitutions for (A) 6B.1A.5b, (B) 6B.1A.5a.1, and (C) 6B.1A.5a.2 viruses. Antigenic sites are shown in different colors, and clade‐defining substitutions are shown in black. Illustrations were created with the PyMol Molecular Graphics System [39].

We carried out experiments to antigenically characterize the 6B.1A.5b, 6B.1A.5a.1, and 6B.1A.5a.2 clades of H1N1 virus found in the NP swab samples. Viruses in the 6B.1A.5b, 6B.1A.5a.1, and 6B.1A.5a.2 clades have multiple HA substitutions relative to the 2019–2020 H1N1 vaccine strain (a 6B.1A.1 virus) (Figure 3 and Table 1). The 6B.1A.5b clade viruses have K160M and K130N substitutions near the Sa antigenic site, a T216K substitution near the Sb antigenic site, and an E235D substitution within the Ca1 antigenic site (Figure 3A). The 6B.1A.5a.1 clade viruses have Q189E and D187A substitutions within the Sb antigenic site, a T185I substitution near the Sb antigenic site, and a N129D substitution near the Ca2 antigenic site (Figure 3B). The 6B.1A.5a.2 viruses have an N156K substitution in the Sa antigenic site, L16II, N129D, and K130N substitutions near the Sa antigenic site, and a T185I substitution near the Sb antigenic site (Figure 3C).

**FIGURE 4:**
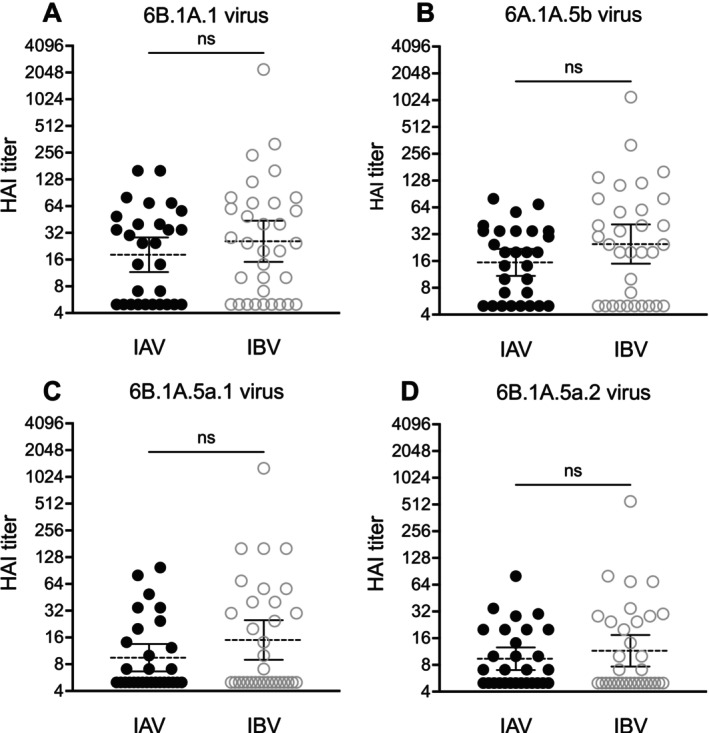
Comparison of antibody titers using sera from IAV‐ and IBV‐infected individuals. HAI assays were completed using sera from IAV‐ and IBV‐infected individuals and 6B.1A.1, 6B.1A.5b, 6B.1A.5a.1, and 6B.1A.5a.2 viruses. Logistic regression analyses using log_2_ geometric mean titers from two independent experiments were completed. For each scatter dot plot, the geometric mean is shown by the dashed line, and 95% confidence intervals are located above and below this line. Data shown are representative of two independent experiments.

**FIGURE 5:**
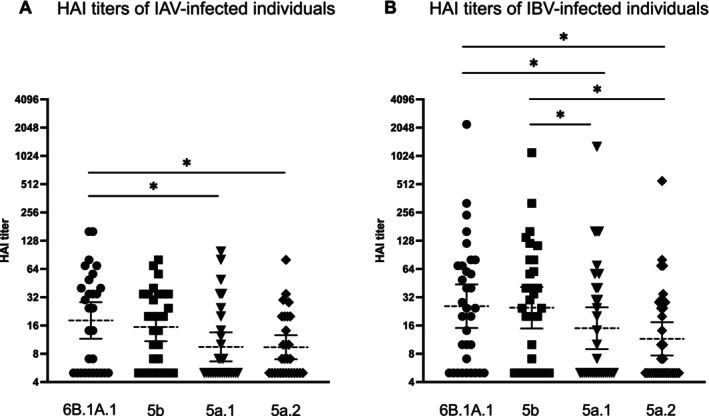
Comparison of antibody titers against different H1N1 clades. HAI assays were completed using sera from (A) H1N1‐infected and (B) IBV‐infected individuals. 6B.1A.1, 6B.1A.5b, 6B.1A.5a.1, and 6B.1A.5a.2 viruses HAI titers were compared by one‐way ANOVA with Dunn's multiple comparisons. Asterisks highlight comparisons where the rank sum difference of HAI titers is significant (*p* < 0.05). Data shown are representative of two independent experiments.

We used reverse genetics to create viruses that possessed an HA from the 6B.1A.1 clade vaccine strain (A/Brisbane/02/2018), a 6B.1A.5b clade virus (A/Delaware/37/2019), a 6B.1A.5a.1 clade virus (A/Hawaii/70/2019), and a 6B.1A.5a.2 clade virus (A/Victoria/2570/2019). We completed HAI assays with each virus and serum samples from H1N1‐ and IBV‐infected individuals. HAI assays detect antibodies that block influenza virus attachment to RBCs [40]. Seven serum samples (24%) from H1N1‐individuals and six samples (18%) from IBV‐infected individuals did not possess detectable levels of antibodies against any of the viruses tested. Antibody titers against each virus were slightly higher in sera from IBV‐infected individuals relative to IAV‐infected individuals, but this did not reach statistical significance using a logistic regression analysis (Figure 4A–D). However, we found significant differences when we compared HAI titers against the 6B.1A.1 vaccine strain and the 6B.1A.5a.1 and 6B.1A.5a.2 clades that dominated the 2019–2020 season (Figure 5; one‐way ANOVA with Dunn's multiple comparisons, *p* < 0.05). HAI titers were higher against the 6B.1A.1 vaccine strain relative to the 6B.1A.5a.1 and 6B.1A.5a.2 clades in H1N1‐infected (Figure 5A) and IBV‐infected (Figure 5B) individuals. HAI titers were similar against the 6B.1A.1 vaccine strain and the less dominant 6B.1A.5b virus using serum from H1N1‐infected individuals (Figure 5A) and IBV‐infected individuals (Figure 5B). Taken together, these data indicate that the 6B.1A.5a.1 and 6B.1A.5a.2 clades, but not the 6B.1A.5b clade, were antigenically distinct relative to the 6B.1A.1 vaccine strain from the 2019–2020 season.

### 3.3 Comparison of Serum From Individuals Infected With Each H1N1 Clade

**FIGURE 6:**
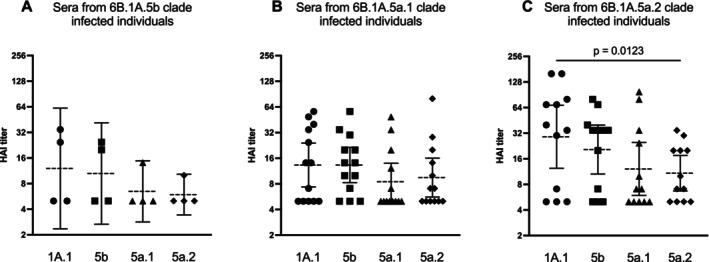
Comparison of HAI titers in sera from individuals infected with different H1N1 clades. HAI data were grouped based on the viral clade detected in each individuals' NP swab. HAI titers against the vaccine strain and circulating viruses were compared by one‐way ANOVA with Dunn's multiple comparisons (*p* < 0.05). For each scatter dot plot, the geometric mean is shown by the dashed line, and 95% confidence intervals are located above and below this line. Data shown are representative of two independent experiments.

Given our observation that 6B.1A.5a.1 and 6B.1A.5a.2 clades were antigenically distinct compared to the 2019–2020 6B.1A.1 vaccine strain, we next compared HAI titers in serum from individuals infected with each unique H1N1 clade to determine whether the individuals infected with circulating viruses had lower antibody levels against their virus of infection. We found that individuals infected with a 6B.1A.5b or 6B.1A.5a.1 virus did not have significant differences in titers against the four viruses tested, although the 6B.1A.5a.1‐infected individuals did trend towards having the lowest mean titer against the 6B.1A.5a.1 virus they were infected with (Figure 6A,B). Similar to the trend that we found with 6B.1A.5a.1‐infected individuals, the 6B.1A.5a.2‐infected individuals had significantly lower mean titers against the 6B.1A.5a.2 virus that they were infected with (*p* = 0.0123) as compared to the 6B.1A.1 H1N1 vaccine strain (Figure 6C; one‐way ANOVA with Dunn's multiple comparisons). Although the relatively low sample numbers in our study prevent firm conclusions with respect to clade‐specific infections, these data suggest that lower antibody reactivity to 6B.1A.5a.2 and potentially 6B.1A.5a.1 viruses facilitated circulation of these H1N1 clades during the 2019–2020 influenza season.

## 4 Conclusions

In this study, we found that influenza virus‐infected individuals seeking care in our health system during the 2019–2020 season possessed antibodies that reacted poorly to the two most prevalent H1N1 clades that circulated. Most individuals in our study were infected with either the 6B.1A.5a.1 or 6B.1A.5a.2 H1N1 clades that dominated circulation in the United States and worldwide [5]. Our experiments indicate that both H1N1 clades are antigenically novel, and we observed the largest amount of antibody reduction against 6B.1A.5a.2 H1N1 viruses. It is notable that most of the 6B.1A.5a.2 clade virus infections occurred later in the season, which correlated with a late season decrease in vaccine effectiveness in the United States [11].

6B.1A.5a.2 viruses possessed a N156K substitution in the Sa antigenic site of HA that likely contributed to reduced antibody reactivity to these viruses. Viruses with the N156K HA substitution first emerged in 2019, and this substitution is now ubiquitously present in H1N1 viruses that continue to circulate in 2024 [5]. Conversely, some of the substitutions observed in our study are no longer present in contemporary circulating H1N1 viruses. For example, the HA1 D187A substitution that was present in 6B.1A.5a.1 viruses was no longer detected at appreciable levels by 2023; however, the Q189E substitution also present in this clade of viruses was found in most viruses circulating in 2024 [5].

A better understanding of the antigenic properties of circulating influenza viruses will improve viral forecasting and the selection of antigenically matched vaccine strains. Although we focused on antigenic analyses in our study, it is also important to evaluate virological properties of influenza virus clades. Recent studies reported that 6B.1A.5a.2 clade viruses replicate less efficiently *in vitro* compared to 6B.1A.5a.1 and 6B.1A.1 viruses [41]. It is possible that the important antigenic changes in 6B.1A.5a.2 clade have allowed the widespread circulation of these viruses despite the observation that these viruses replicate less efficiently compared to other H1N1 clades. It is also possible that 6B.1A.5a.2 viruses replicate more efficiently in the human upper airways and that this is not captured using *in vitro* assays.

Our study has some limitations. We only tested samples from one site in Philadelphia, and the relatively small sample size did not allow us to reach firm statistically significant conclusions for some comparisons. For example, we found that IBV‐infected individuals had higher levels of antibodies against the 6B.1A.5b clade and the 6B.1.A.1 vaccine strain clade relative to H1N1‐infected individuals, but this did not reach statistical significance. Moving forward, we will expand our sequencing efforts and antigenic testing of a larger number of samples collected at HUP in upcoming influenza seasons. Additional antigenic analyses evaluating infections at the clade level will improve our understanding of human immunity against influenza viruses and ultimately improve viral forecasting and the development of improved influenza vaccines.

## Author Contributions

**Lydia M. Mendoza:** conceptualization, investigation, writing – original draft, formal analysis. **Elizabeth M. Anderson:** investigation. **Ashley Sobel Leonard:** investigation. **Alexander G. McFarland:** investigation. **Jordan T. Ort:** investigation. **Nicole Tanenbaum:** investigation. **Afeesat Durosinmi:** investigation. **Frederic D. Bushman:** supervision. **Laurel J. Glaser:** supervision. **Irving Nachamkin:** supervision. **Scott E. Hensley:** supervision, funding acquisition, writing – review and editing, conceptualization.

## Ethics Statement

All experiments involving samples from human subjects was reviewed and approved by the University of Pennsylvania Institutional Review Board (Protocol No. 834238).

## Consent

Leftover deidentified clinical samples were used in this study.

## Conflicts of Interest

S.E.H. is a co‐inventor on patents that describe the use of nucleoside‐modified mRNA as a vaccine platform. S.E.H. reports receiving consulting fees from Sanofi, Pfizer, Lumen, Novavax, and Merck.

### Peer Review

The peer review history for this article is available at https://www.webofscience.com/api/gateway/wos/peer‐review/10.1111/irv.70104.

## Supporting information


**Table S1.** List of the laboratories contributing to sequences from GISAID’s EpiFlu database. We gratefully acknowledge the authors, originating and submitting laboratories of the sequences from GISAID’s EpiFlu Database. All submitters of data may be contacted directly via www.gisaid.org.

## Data Availability

All data and code are included in this manuscript and on referenced GitHub sites.
